# Polyethyleneimine-Oleic Acid Micelles-Stabilized Palladium Nanoparticles as Highly Efficient Catalyst to Treat Pollutants with Enhanced Performance

**DOI:** 10.3390/polym13111890

**Published:** 2021-06-06

**Authors:** Xiang Lai, Xuan Zhang, Shukai Li, Jie Zhang, Weifeng Lin, Longgang Wang

**Affiliations:** 1 Key Laboratory of Applied Chemistry, Hebei Key Laboratory of Heavy Metal Deep-Remediation in Water and Resource Reuse, College of Environmental and Chemical Engineering, Yanshan University, Qinhuangdao 066004, China; lx0406x@163.com (X.L.); 15603395687@163.com (X.Z.); lishukai1998@163.com (S.L.); 19991641458@163.com (J.Z.); 2 Department of Molecular Chemistry and Materials Science, Weizmann Institute of Science, Rehovot 76100, Israel; lin.weifeng@weizmann.ac.il

**Keywords:** polyethyleneimine, micelles, palladium, nanoparticles, catalytic

## Abstract

Water soluble organic molecular pollution endangers human life and health. It becomes necessary to develop highly stable noble metal nanoparticles without aggregation in solution to improve their catalytic performance in treating pollution. Polyethyleneimine (PEI)-based stable micelles have the potential to stabilize noble metal nanoparticles due to the positive charge of PEI. In this study, we synthesized the amphiphilic PEI-oleic acid molecule by acylation reaction. Amphiphilic PEI-oleic acid assembled into stable PEI-oleic acid micelles with a hydrodynamic diameter of about 196 nm and a zeta potential of about 34 mV. The PEI-oleic acid micelles-stabilized palladium nanoparticles (PO-PdNPs_n_) were prepared by the reduction of sodium tetrachloropalladate using NaBH_4_ and the palladium nanoparticles (PdNPs) were anchored in the hydrophilic layer of the micelles. The prepared PO-PdNPs_n_ had a small size for PdNPs and good stability in solution. Noteworthily, PO-PdNPs_150_ had the highest catalytic activity in reducing 4-nitrophenol (4-NP) (*K_nor_* = 18.53 s^−1^mM^−1^) and oxidizing morin (*K_nor_* = 143.57 s^−1^M^−1^) in aqueous solution than other previous catalysts. The enhanced property was attributed to the improving the stability of PdNPs by PEI-oleic acid micelles. The method described in this report has great potential to prepare many kinds of stable noble metal nanoparticles for treating aqueous pollution.

## 1. Introduction

The quality of water is highly related with our health. Many countries have strictly controlled the emissions of various organic pollutants in water [1]. For example, 4-nitrophenol (4-NP) is highly toxic, but its reduced product 4-aminophenol (4-AP) is relatively low in toxicity and is a pharmaceutical intermediate. The catalytic reduction of 4-NP and similar phenol compounds is carried out on catalysts treated with NaBH_4_. In addition, morin is a kind of polyphenol dye that belongs to flavonoid dyes. Morin has been used as a model matrix for the study of catalytic bleaching processes in laundry detergents [2,3]. Morin can be degraded by using nanoparticles with H_2_O_2_. These nanoparticles play an important role for the catalytic generation of reactive oxygen species from decomposition of H_2_O_2_. Thus, the efficiency of treatment of organic pollutants is highly dependent on the property of catalysts.

Many kinds of noble metal nanoparticles, such as platinum nanoparticles (PtNPs) [4], gold nanoparticles (AuNPs) [5,6], and palladium nanoparticles (PdNPs) [7,8,9], catalyze the reduction of 4-NP and the oxidation of morin. The catalytic activity of noble metal nanoparticles is highly dependent on the active atoms on their surface, which results in extremely high surface energy of the nanoparticles [10,11]. However, it is for this reason that the nanoparticles are easily agglomerated in the preparation and catalytic reaction process [12], which results in a significant decrease in the number of surface-active atoms and the catalytic activity. This problem limits noble metal nanoparticles in practical applications [13]. Researchers have developed a variety of stabilizers to prevent the coagulation of noble metal nanoparticles, thereby increasing their catalytic efficiency [14,15]. For example, Pitchaimani Veerakumar and his colleagues report a method for immobilizing PdNPs (Pd/NH_2_-SiO_2_) with PEI (Mw = 25,000) functionalized silica nanoparticles that have good separation properties and exhibit excellent catalytic performance [16]. As a kind of polymer, PEI has a good advantage as a stabilizer. Each unit with three atoms in the PEI skeleton has a nitrogen atom [17]. Since PEI has a positive charge, it generates electrostatic attraction with the negatively charged noble metal precursor [18], thereby stabilizing the noble metal nanoparticles. However, it is difficult for the low-molecule-weight PEI to stabilize the noble metal nanoparticles. The micelles formed by amphiphilic molecules based on the reaction of low-molecule-weight PEI with other hydrophobic molecules can be one method to stabilize noble metal nanoparticles and solve the shortcomings of low-molecule-weight PEI.

Herein, PEI-oleic acid micelle-stabilized PdNPs (PO-PdNPs_n_) were prepared by using PEI-based micelles. The amphiphilic molecule consisted of low-molecule-weight PEI (Mw = 600) as hydrophilic moiety and oleic acid as hydrophobic moiety. PEI and oleic acid are also inexpensive and readily available. The prepared PdNPs were small in size and had a narrow size distribution. The PO-PdNPs_n_ showed high stability and an enhanced catalytic efficiency to treat pollutants such as 4-NP and morin. The current work provides new ideas for the synthesis of noble metal nanoparticle catalysts with amphiphilic molecular micelles to treat pollutants.

## 2. Materials and Methods

### 2.1. Preparation of PEI-Oleic Acid Micelles

The synthesis of PEI-oleic acid refers to the method reported in the previous literature [17]. First, 0.60 g PEI, 0.28 g oleic acid, 0.40 g EDC·HCl, 0.27 g HOBt, and 6 mL of anhydrous dimethyl formamide were added into a flask and passed through with N_2_ for 0.5 h. The obtained solution was dialyzed against methanol with a dialysis bag (MWCO = 500) after 1 day. Then, the PEI-oleic acid was obtained by the removal of methanol with the help of a rotary evaporator (RE-52A, Shanghai Yarong Biochemical Instrument Factory, Shanghai, China). PEI-oleic acid in methanol solution was added dropwise to water (methanol: H_2_O = 1:9) at 25 °C for 10 min. The PEI-oleic acid micelles solution was obtained by dialysis.

### 2.2. Preparation of PO-PdNPs_n_

Two mM Na_2_PdCl_4_ and the PEI-oleic acid micelles were mixed. The molar ratios of N atoms of the PEI-oleic acid micelles to Pd atoms of Na_2_PdCl_4_ were 75, 100, and 150, respectively. After 20 min, a 5-fold molar excess of NaBH_4_ in 0.3 M NaOH was added. One M HCl was added to tune the pH to neutral after 20 min. The mixed solution reacted for 1 h to obtain PO-PdNPs_n_ (*n* = 75, 100, 150).

### 2.3. Critical Micelles Concentration Measurement

The critical micelle concentration of the PEI-oleic acid micelles in aqueous solution was determined by a pyrene fluorescence probe [19]. Briefly, the fluorescence intensity values of 374 nm and 384 nm at the excitation wavelength of 334 nm were measured by fluorescence spectrophotometer. The CMC value of the PEI-oleic acid micelles in water was determined by the ratio of fluorescence intensity at 374 nm and 384 nm.

### 2.4. Catalytic Reaction on 4-NP

To study the degradation of 4-NP over time, 250 µL of 4-NP solution (600 µM), 1 mL of water, and 1 mL of fresh NaBH_4_ solution (0.5 M) were added in a quartz cuvette (1 × 1 cm^2^) in sequence at 25 °C. Then, 50 µL of PO-PdNPs_75_ (8.35 µM) was added. UV–Vis spectra within 200–800 nm of the mixed solution were recorded every 3 min.

To explore the relationship between PO-PdNPs_n_ and the catalytic rate, 250 µL of 4-NP (600 µM) solution, water, and 1 mL of fresh NaBH_4_ solution (0.5 M) were added into a quartz cuvette followed by the addition 50 μL of PO-PdNPs_n_ (*n* = 75, 100, 150), respectively. The absorbance at 400 nm was measured by UV-TU1810 (Beijing Purkinje General Instrument Co., Ltd., Beijing, China).

### 2.5. Catalytic Reaction on Morin

Eighty μL of morin in a carbonate buffer at pH 9.2 (3 mM), 1790 μL of carbonate buffer at pH 9.2, 50 μL of PO-PdNPs_75_ (0.475 mM), and 80 μL of hydrogen peroxide (0.4 mM) were added to the cuvette. UV–Vis spectra within 200–800 nm of the mixed solution were recorded every 2 min. As the same time, the spectra of the control experiment were measured every 5 min.

The effects of different catalyst concentrations and different morin concentrations on the catalytic reaction were determined by UV-TU1810 (Beijing Purkinje General Instrument Co., Ltd., Beijing, China) at 403 nm. One mM morin, carbonate buffer, PO-PdNPs_n_ (*n* = 75, 100, 150), and 0.4 M hydrogen peroxide were added to a cuvette. The final concentration of [N] in PdNPs_n_ and hydrogen peroxide were 7.8 mM and 10 mM, respectively. The concentration of morin was from 0.5 to 1.25 mM. The next experiment had similar procedure. The concentration of [N] in the catalyst was fixed at 0.78–5.47 mM with 3 mM morin.

### 2.6. Statistical Analysis

The *k*_app_ data of the PO-PdNPs_n_ of 4-NP and the morin treatment were analyzed by SPSS 25 to assess the statistical differences between the groups. *p* < 0.05 is considered statistically significant.

## 3. Results

### 3.1. Characterization of PEI-Oleic Acid Micelles

**Figure 1:**
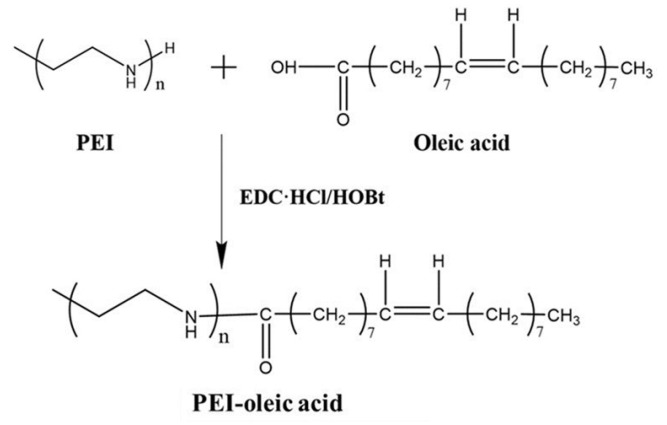
Synthesis of amphiphilic molecule PEI-oleic acid.

Acylation reaction was employed into the synthesis of the amphiphilic molecules of PEI-oleic acid, which is illustrated in Figure 1. In this reaction, PEI (Mw = 600) and oleic acid had the same amount, EDC·HCl and HOBt acted as coupling reagents in dry DMF at room temperature for 24 h in the acylation reaction. The prepared PEI-oleic acid amphiphilic molecule had PEI as the hydrophilic segment and oleic acid as the hydrophobic segment.

The PEI-oleic acid amphiphilic molecules self-assembled in water to form micelles, and the hydrophilic PEI segment acted as a shell layer of the micelles. The critical micelle concentration (CMC) of the PEI-oleic acid micelles was [N] = 0.12 mg/mL based on the fluorescence method using pyrene. The hydrodynamic diameter and zeta potential of PEI-oleic acid micelles were determined to be 196 nm and 34 mV by DLS (DLS, Malvern, Worcestershire, UK), respectively. The positive charge of the micelles was attributed to the hydrophilic PEI shell.

### 3.2. Characterization of PO-PdNPs_n_

**Figure 2:**
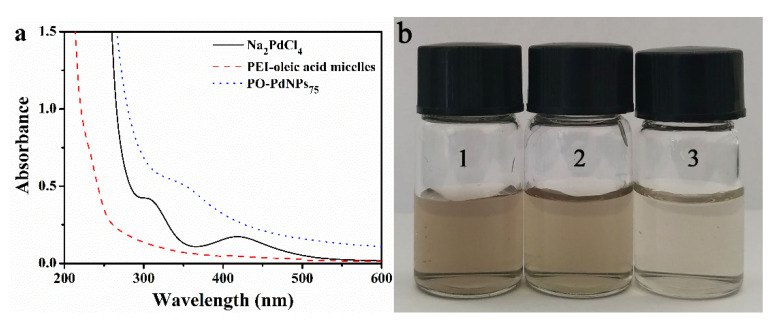
(**a**) UV–Vis spectra of Na_2_PdCl_4_, PEI-oleic acid micelles, and PO-PdNPs_75_, (**b**) PO-PdNPs_n_ with molar ratio of *n* = [N]: [Pd] = 75 (**b1**), 100 (**b2**), and 150 (**b3**), respectively.

To prepare PO-PdNPs_n_, Na_2_PdCl_4_ was mixed with PEI-oleic acid micelles and then further reduced with NaBH_4_. As shown in Figure 2a, two characteristic absorption peaks at 305 and 420 nm were detected for the Na_2_PdCl_4_. After the reduction of Na_2_PdCl_4_ by NaBH_4_ to produce PdNPs, a new absorption spectrum appeared, which indicated the formation of PdNPs. This was consistent with the results reported in the previous literature [20]. As the molar ratio of [N]:[Pd] ranged from 75 to 150, the color of solution gradually decreased as shown in Figure 2b. The UV–Vis spectra and color change of solution indicated that the PdNPs were successfully stabilized by the PEI-oleic acid micelles. The positively charged PEI shell played an important role in adsorbing and stabilizing PdNPs. As shown in Figure S1 and Table S1, the infrared spectroscopy has shown the successful preparation of PEI-oleic acid micelles and PO-PdNPs_75_.

**Figure 3:**
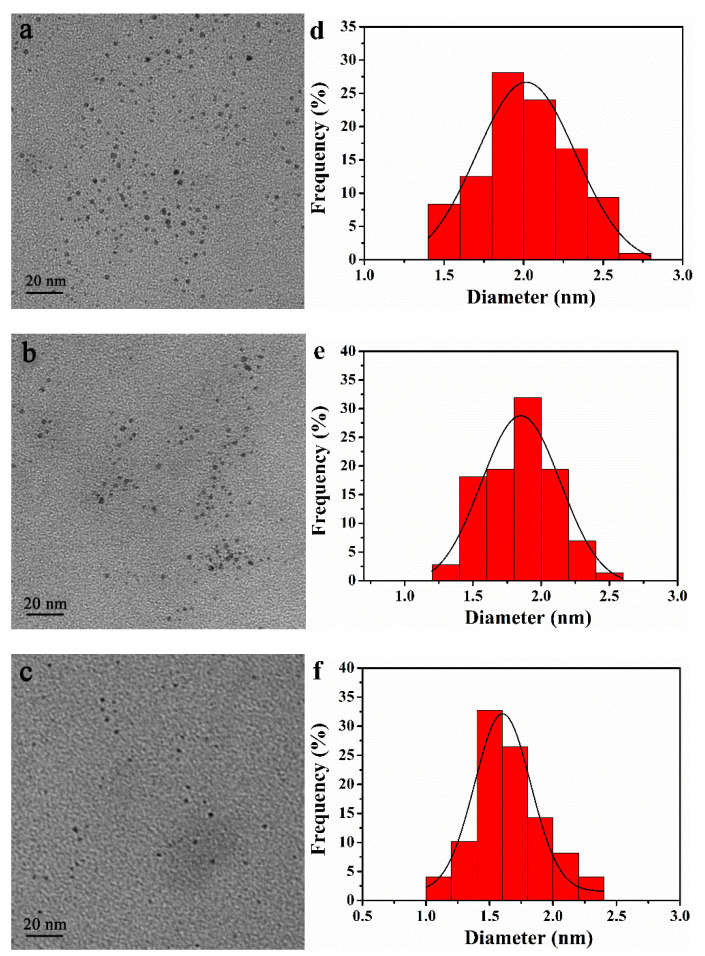
TEM images and size distribution analysis of the PdNPs of PO-PdNPsn: (**a**,**d**) *n* = 75, (**b**,**e**) *n* = 100, (**c**,**f**) *n* = 150.

The size of the PdNPs of PO-PdNPs_n_ was measured by TEM (TEM, JEM-1230EX, Hitachi, Tokyo, Japan). Figure 3 displays the monodispersed characteristic of PO-PdNPs_n_. The mean particle sizes of PdNPs within PO-PdNPs_n_ at [N]:[Pd] = 75, 100, and 150 were 2.01 ± 0.30, 1.85 ± 0.25, and 1.67 ± 0.27 nm, respectively. The size of the PdNPs decreased with the increasing the molar ratio of [N]:[Pd]. In short, the prepared PdNPs of PO-PdNPs_n_ had a small particle size and a narrow size distribution.

**Figure 4:**
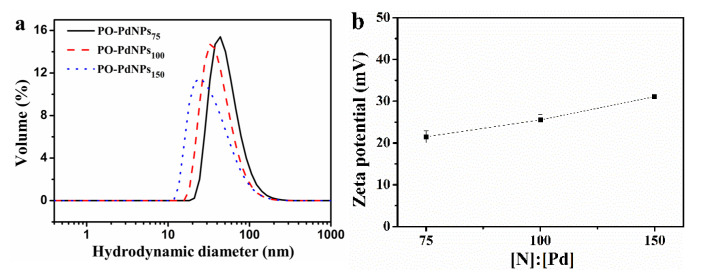
(**a**) Hydrodynamic diameter and (**b**) zeta potential of PO-PdNPs_n_.

In order to determine the stability of the prepared PO-PdNPs_n_ in aqueous solution, the hydrodynamic diameter and zeta potential were measured by DLS (DLS, Malvern, Worcestershire, UK) [21,22]. As shown in Figure 4a, the hydrodynamic diameters were about 53.89, 43.95, and 37.86 nm at a molar ratio of [N]:[Pd] = 75, 100, and 150, respectively. These hydrodynamic diameters of the prepared PO-PdNPs were smaller than that of the PEI-oleic acid micelles, which should be attributed to PdNPs located in the PEI-oleic acid micelles’ shell. The measured hydrodynamic diameters of the prepared PO-PdNPs was larger than the TEM particle size measured in the dry state [23,24]. Figure 4b showed that the zeta potential of PO-PdNPs_n_ was 21.5, 25.55, and 31.1 mV at a molar ratio of [N]:[Pd] = 75, 100, and 150, respectively. The positive charge on the surface of PO-PdNPs_n_ was larger than 20 mV. Thus, the strong electrostatic repulsion was good for the high stability of PO-PdNPs_n_ in aqueous solution. In summary, PO-PdNPs_n_ had high stability in solution and big positive charge, so it kept from coagulation within one month.

### 3.3. Catalytic Activity of PO-PdNPs_n_ on 4-NP

**Figure 5:**
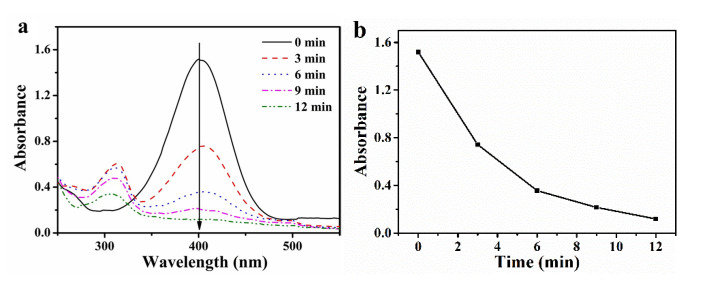
(**a**) The UV–Vis spectra of the reduction process of 4-NP catalyzed by PO-PdNPs_75_, (**b**) the relationship diagram of absorbance and time.

The catalytic reduction of 4-NP was employed to evaluate the catalytic activity of PO-PdNPs_n_. Due to the high toxicity of 4-NP and its pollution of water resources, it has attracted great attention. 4-AP is the reduction product of 4-NP. 4-AP has low toxicity and important applications in industry. It is important to study the method to transform 4-NP into 4-AP. The concentration of PdNPs in the solution of the reacting mixture was as low as 10^−7^ mM. Therefore, the absorption spectra of PdNPs almost had no effect on the absorbance of the mixed solution. The absorption peak of 4-NP existed at 317 nm. When the NaBH_4_ solution was added, the color of the solution rapidly turned yellow, and the obvious absorption peak appeared at 400 nm, which corresponded to 4-hydroxyaminophenol [25]. In Figure 5a, it was found that the absorption peak at 400 nm decreased gradually, and the absorption peak at 310 nm increased. The results indicated the formation of 4-AP due to the consumption of 4-hydroxyaminophenol. After 12 min, the color of the solution became colorless, and the absorption peak at 400 nm decreased to 0.119 as shown in Figure 5b, indicating that 4-NP completely changed to 4-AP. The turnover frequency (TOF) was defined as the number of reactants converted per h. The TOF of PO-PdNPs_75_ reached 1796.4 h^−1^ in this experiment.

**Figure 6:**
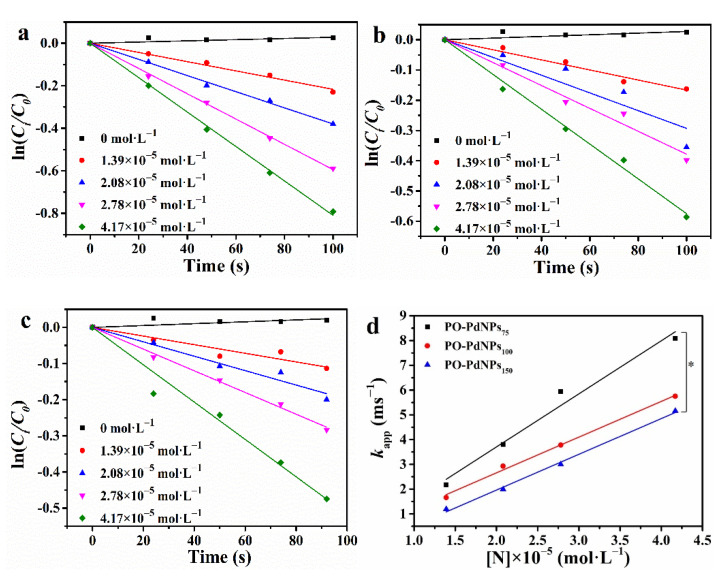
The catalytic process for ln(*C_t_/C*_0_) and time (**a**) PO-PdNPs_75_, (**b**) PO-PdNPs_100_, (**c**) PO-PdNPs_150_, (**d**) the linear relationship between *k*_app_ and concentration of PO-PdNPs_n_. * *p* < 0.05.

As shown in Figure 6a–c, PO-PdNPs_n_ (*n* = 75, 100, and 150) had a linear relationship between ln (*C_t_/C*_0_) and reaction time (t). Thus, the catalytic reduction of 4-NP by using PO-PdNPs_n_ followed pseudo-first-order kinetics. This was attributed to excess NaBH_4_ in the mixed solution. Thus, the apparent rate constant (*k*_app_) was calculated as follows:

(1) $$\frac{dC_{t}}{dt}=\ln(C_{t}/C_{0})=-k_{app}t$$

**Figure 7:**
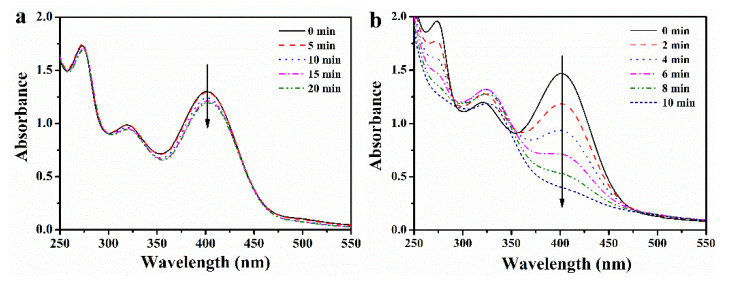
The UV–Vis spectra of the oxidation of morin (**a**) without the catalyst every 5 min and (**b**) with the PO-PdNPs_75_ every 2 min.

The *k*_app_ values were calculated from the slope of the lines as shown in Figure 7. The corresponding *k*_app_ was shown in Figure 6d. The *k*_app_ value increased with increasing catalyst concentration of PO-PdNPs_n_, indicating that the reduction rate was linear correlated with the concentration of PO-PdNPs_n_. *K_nor_* was used to compare the catalytic activity of different catalysts, *K_nor_* was defined as the ratio of *k*_app_ to molar concentration of catalyst (*K_nor_* = *k*_app_/*C_cat_*). PO-PdNPs_150_ had the highest *K_nor_* (18.53 s^−1^mM^−1^) in PO-PdNPs_n_. Table 1 shows the *K_nor_* and the TOF of the PO-PdNPs_n_ and other catalysts. PO-PdNPs_150_ had the highest *K_nor_*, indicating that they had the highest activity. PdNPs were located in the micelles’ shells, which may contribute to their high catalytic activity caused by the low mass transfer resistance of the substrates. In short, this catalytic reaction followed pseudo-first-order kinetics and PO-PdNPs_150_ had the highest activity.

### 3.4. Catalytic Activity of PO-PdNPs_n_ on Morin

Morin is a polyphenol dye that has been used as a model to study the catalytic ability of precious metal nanoparticle catalysts. The catalytic activity and catalytic mechanism of PO-PdNPs_n_ were evaluated with the addition of H_2_O_2_ [29,30]. In a carbonate buffer with pH 9.2, the maximum peak of the morin was λ = 403 nm. Figure 7a shows that without the addition of a catalyst, the maximum peak of morin hardly decreased within 20 min with H_2_O_2_. Figure 7b shows that after adding the catalyst PO-PdNPs_75_, the absorbance at 403 nm decreased rapidly with time, while the absorbance at 325 nm gradually increased with time. This phenomenon indicated that the morin underwent catalyzed oxidation to benzofuranone [31]. After a longer time, the peak at 325 nm decreased again, indicating that benzofuranone was further oxidized [32,33]. Therefore, the experimental study of the reaction kinetics was carried out by controlling the reaction time.

**Figure 8:**
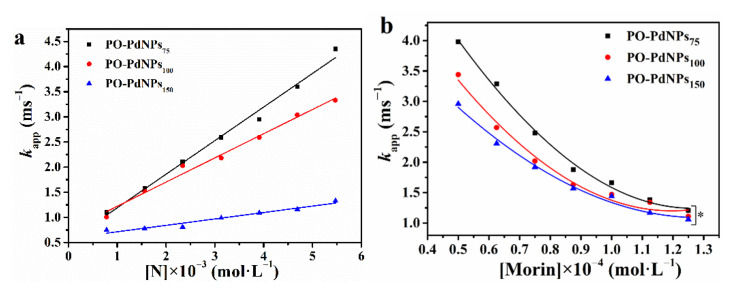
(**a**) The effect of different concentration and ratio of catalysts on the catalytic reaction *k*_app_. (**b**) The effect of different concentrations of morin on the catalytic reaction *k*_app_ (C_H2O2_ = 10 mM, C_[N]_ = 0.78 mM). * *p* < 0.05.

To study the relationship between *k*_app_ and PO-PdNPs_n_ concentration, the effect of changing the concentration of PO-PdNPs_n_ on the catalytic reaction under the conditions of constant concentration of morin and H_2_O_2_ was measured. Figure 8a showed that the relationship between *k*_app_ and the catalyst concentration was linear. With the increase of catalyst concentration, *k*_app_ became higher. The three proportions of catalysts had the same trend as follows: PO-PdNPs_75_ > PO-PdNPs_100_ > PO-PdNPs_150_. The *K_nor_* of PO-PdNPs_75_, PO-PdNPs_100_, and PO-PdNPs_150_ were 105.77, 129.49, and 143.57 s^−1^M^−1^, respectively, so the catalytic activity was increased according to the order of PO-PdNPs_75_ <PO-PdNPs_100_ <PO-PdNPs_150_. This was because the particle size of PdNPs in PO-PdNPs_n_ decreased in the order of PO-PdNPs_75_ <PO-PdNPs_100_ <PO-PdNPs_150_. At the same concentration, the smaller particle size resulted in the larger specific surface area and the higher catalytic efficiency.

To study the relationship between *k*_app_ and the morin concentration, this experiment monitored the process of morin reaction by fixing the H_2_O_2_ concentration during the reaction process. As shown in Figure 8b, *k*_app_ decreased with increasing morin concentration. This was consistent with the previously reported results of catalytic oxidation of morin by MnOx [34]. Surface coverage was the primary factor of the reaction rate that was dependent on the different concentration and adsorption constant of morin and H_2_O_2_. Generally, during the reaction of morin, the adsorption constant *K_morin_* is higher than *K*_H2O2_. Therefore, compared with H_2_O_2_, morin is more easily adsorbed on the catalyst surface. As the concentration of morin increased, the active sites on the surface of noble metal nanoparticles are covered, resulting in a decrease in the active sites available for H_2_O_2_ adsorption, which gradually decreases the reaction rate when the concentration of morin increases.

### 3.5. Catalytic Mechanism

During the catalytic reduction of 4-NP, the Langmuir–Hinshelwood kinetics method was followed according to previous reports [35,36]. Similarly, the catalytic reduction of 4-NP by PO-PdNPs_n_ should also meet the equation. 4-NP was quickly reduced to a stable intermediate 4-hydroxyaminophenol. The electrons from sodium borohydride and 4-hydroxyaminophenol ions combined on the surface of PdNPs to produce 4-AP ions, and then 4-AP ions were desorbed to form 4-AP molecules. In this process, the adsorption/desorption equilibria of all related compounds on the surface of PdNPs were rapidly achieved. In the oxidation process of morin by PO-PdNPs_n_, the Langmuir –Hinshelwood kinetics method was also followed [29,37].

## 4. Conclusions

In conclusion, PEI-oleic acid micelles were prepared and used to stabilize PdNPs in aqueous solution. The size of PdNPs in the PO-PdNPs_n_ was between 1.67 and 2.01 nm with a narrow size distribution. The PO-PdNPs_n_ maintained high stability due to its zeta potentials larger than 20 mV. In addition, the prepared PO-PdNPs_n_ effectively catalyzed the reduction of 4-NP to form 4-AP. PO-PdNPs_n_ also had high catalytic efficiency for morin. The catalytic ability of PO-PdNPs_n_ was higher than those of other catalysts, which could result from the small size and high stability of Pd NPs. The location of PdNPs in the PEI-oleic acid micelles was good for low mass transfer resistance. The prepared PO-PdNPs_n_ has great potential applications in treating various organic contaminants in solution in the future.

## Figures and Tables

**Table 1:** Comparison of *K_nor_* and TOF of PO-PdNPs_n_ with other Pd-based catalysts in 4-NP reduction.

Catalyst	*K*_nor_ (s^−1^mM^−1^)	TOF (h^−1^)	Reference
PdP/CNSs	1.4	504	[2]
Pd/Fe_3_O_4_@SiO_2_@KCC-1	2.78	-	[26]
Pd/SBA-15	0.118	-	[27]
@Pd/CeO_2_	-	1068	[28]
PO-PdNPs_75_	-	1796	This work
PO-PdNPs_150_	18.53	-	This work

## Data Availability

The data presented in this study are available on request from the corresponding author.

## Supplementary Materials

The following are available online at https://www.mdpi.com/article/10.3390/polym13111890/s1, Figure S1: FTIR spectra of PEI-oleic acid micelles and PO-PdNPs_75_, Table S1: IR bands of the two compounds and their assignments.
