# Supplementary material for: Polyethyleneimine-Oleic Acid Micelles-Stabilized Palladium Nanoparticles as Highly Efficient Catalyst to Treat Pollutants with Enhanced Performance
Source: Polymers (Basel). 2021 Jun 6;13(11):1890. doi: 10.3390/polym13111890 (PMC8201335; doi:10.3390/polym13111890)
Supplement: Supplementary file 1 [file polymers-13-01890-s001.zip › polymers-1220837-supplementary.pdf]

Supplementary Material

Polyethyleneimine-Oleic Acid Micelles-Stabilized Palladium Nanoparticles as Highly Efficient Catalyst to Treat Pollutants with Enhanced Performance

Xiang Lai[1†], Xuan Zhang[1†], Shukai Li[1], Jie Zhang[1], Weifeng Lin[2], Longgang Wang[1]*

[1.] *Key Laboratory of Applied Chemistry, Hebei Key Laboratory of heavy metal deep-remediation in water and resource reuse, College of Environmental and Chemical Engineering, Yanshan University, Qinhuangdao, 066004, China*

[2] *Department of Molecular Chemistry and Materials Sciences, Weizmann Institute of Science, Rehovot, 76100, Israel*

† *These authors contributed equally to this work.*

2. Materials and Characterization

2.1 Materials

Polyethyleneimine (Mw=600), sodium tetrachloropalladate (Na_2PdCl_4), H_2O_2, sodium borohydride ($NaBH_4$), 4-nitrophenol (4-NP) and morin were purchased from Aladdin. 1-hydroxybenzotriazole (HOBt) and pyrene were purchased from Energy Chemical. 1-ethyl-3-(3-dimethylaminopropyl) carbodiimide hydrochloride (EDC·HCl) was purchased from GL Biochem (Shanghai) Ltd. Methanol, N, N-dimethylformamide (DMF) and acetone were purchased from Tianjin Guangfu Technology Development Co. Ltd. Oleic acid, sodium carbonate, sodium bicarbonate, and hydrochloric acid were purchased from Sinopharm Chemical Reagent Beijing Co. Ltd. Dialysis bags with molecular weight cut-off (MWCO=500) were purchased from Spectrum Laboratories Inc.

2.2 Characterization measurement

The size of PdNPs in dry state was measured by transmission electron microscopy (TEM, JEM-1230EX, Hitachi, Tokyo, Japan)). The hydrodynamic diameter and zeta potential of PO-$PdNPs_n$ (1 mg/mL) were measured by dynamic light scattering (DLS, Malvern, Worcestershire, UK). The functional group information of PEI-oleic acid micelles and PO-$PdNPs_n$ was recorded by Fourier transform infrared spectroscopy (FTIR, E55-FRA106, Bruker, Karlsruhe, Germany).

3 Results and discussion

Figure S1 showed FTIR spectra of PEI-oleic acid micelles and PO-$PdNPs_n$. As shown in the spectrum of PEI-oleic acid: (1) The peak at 2920 cm^{-1} is designated as CH stretching vibration, and the bands at 2850 cm^{-1} and 1465 cm^{-1} were attributed to the CH_2 stretching vibration of long fat chain. (2) The peak near 1150 cm^{-1} was designated as C-C stretching vibration; (3) The peak at 1650 cm^{-1} corresponded to the vibration of amide group. This result indicated that PEI reacted with oleic acid to form PEI-oleic acid micelles. In addition, the FTIR spectra of PO-$PdNPs_n$ did not change significantly with PEI-oleic acid micelles, but the intensity of some peaks had changed, which indicated that the structure of PEI-oleic acid micelles changed after loading PdNPs. PEI-oleic acid molecule played an important role in stabilizing PdNPs.

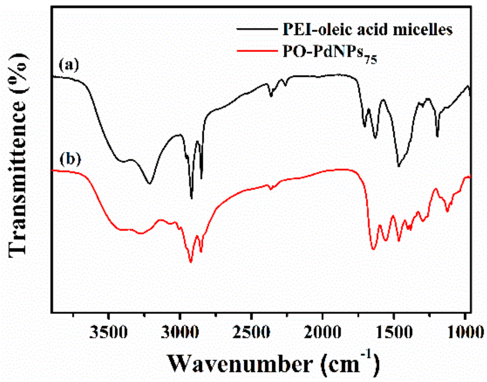

Figure S1. FTIR spectra of PEI-oleic acid micelles and PO-PdNPs$_{75}$

Table S1. IR bands of the two compounds and their assignments

Wavenumber (cm^{-1})	Assignments
2920	CH stretching vibration
2850	CH_2 stretching vibration of long fat chain
1650	the vibration of amide group
1465	CH_2 stretching vibration of long fat chain
1150	C-C stretching vibration
